# Pediatric Measles Vaccine Expressing a Dengue Antigen Induces Durable Serotype-specific Neutralizing Antibodies to Dengue Virus

**DOI:** 10.1371/journal.pntd.0000096

**Published:** 2007-12-12

**Authors:** Samantha Brandler, Marianne Lucas-Hourani, Arnaud Moris, Marie-Pascale Frenkiel, Chantal Combredet, Michèle Février, Hugues Bedouelle, Olivier Schwartz, Philippe Desprès, Frédéric Tangy

**Affiliations:** 1 Viral Genomics and Vaccination Laboratory, Institut Pasteur, CNRS-URA3015, Paris, France; 2 Flavivirus-Host Molecular Interactions Unit, Institut Pasteur, Paris, France; 3 Virus and Immunity Group, Institut Pasteur, CNRS-URA3015, Paris, France; 4 Molecular Prevention and Therapy of Human Diseases Unit, Institut Pasteur, CNRS-URA3012, Paris, France; Oswaldo Cruz Foundation, Brazil

## Abstract

Dengue disease is an increasing global health problem that threatens one-third of the world's population. Despite decades of efforts, no licensed vaccine against dengue is available. With the aim to develop an affordable vaccine that could be used in young populations living in tropical areas, we evaluated a new strategy based on the expression of a minimal dengue antigen by a vector derived from pediatric live-attenuated Schwarz measles vaccine (MV). As a proof-of-concept, we inserted into the MV vector a sequence encoding a minimal combined dengue antigen composed of the envelope domain III (EDIII) fused to the ectodomain of the membrane protein (ectoM) from DV serotype-1. Immunization of mice susceptible to MV resulted in a long-term production of DV1 serotype-specific neutralizing antibodies. The presence of ectoM was critical to the immunogenicity of inserted EDIII. The adjuvant capacity of ectoM correlated with its ability to promote the maturation of dendritic cells and the secretion of proinflammatory and antiviral cytokines and chemokines involved in adaptive immunity. The protective efficacy of this vaccine should be studied in non-human primates. A combined measles–dengue vaccine might provide a one-shot approach to immunize children against both diseases where they co-exist.

## Author Summary

Dengue is a tropical emerging disease that threatens one-third of the world's population, mainly children under the age of 15. The development of an affordable pediatric vaccine that could provide long-term protection against all four dengue serotypes remains a global public health priority. To address this challenge, we evaluated a strategy based on the expression of a minimal dengue antigen by live attenuated measles vaccine (MV), one of the most safe, stable, and effective human vaccines. As a proof-of-concept, we constructed a MV vector expressing a secreted dengue antigen composed of the domain III of the envelope glycoprotein (EDIII), which contains major serotype-specific neutralizing epitopes, fused to the ectodomain of the membrane protein (ectoM) from DV-1, as an adjuvant. This vector induced in mice durable serotype-specific virus-neutralizing antibodies against DV1. The remarkable adjuvant capacity of ectoM to EDIII immunogenicity was correlated to its capacity to mature dendritic cells, known to initiate immune response, and to activate the secretion of a panel of cytokines and chemokines determinant for the establishment of specific adaptive immunity. Such strategy might offer pediatric vaccines to immunize children simultaneously against measles and dengue in areas of the world where the diseases co-exist.

## Introduction

Dengue fever is a mosquito-borne viral disease that threatens the health of a third of the world's population. During the last twenty years, the four serotypes of dengue virus spread throughout the tropics due to the presence of the mosquito vector *Aedes aegypti* in all urban sites and to the major demographic changes that occurred in these regions. This global re-emergence shows larger epidemics associated with more severe disease [1]. Dengue is a major worldwide public health problem with an estimated 100 million annual cases of dengue fever (DF) and 500,000 annual cases of dengue hemorrhagic fever (DHF), the severe form of the disease, resulting in about 25,000 fatal cases, mainly in children under the age of 15. Although global prevention appears the best strategy to control dengue expansion, there is still no licensed vaccine available.

Dengue viruses (DV) are enveloped, positive-stranded RNA viruses (*Flaviviridae* family). Four antigenically distinct viral serotypes exist (DV1-4). The surface of virions is composed of the major envelope glycoprotein (E) and a small membrane protein (M). Very little information is available concerning the role of the 75-amino acid long M protein. We previously reported that ectopic expression of the 40-residue intraluminal ectodomain of M (referred hereafter as ectoM) is able to induce apoptosis in mammalian cells, suggesting that M might play an important role in the pathogenicity of flaviviruses [2]. The envelope E protein, which is exposed on the surface of viral particles, is responsible for virus attachment and virus-specific membrane fusion. Anti-E antibodies inhibit viral binding to cells and neutralize infectivity. A primary DV infection is believed to induce life-long immunity to the infecting serotype, while heterologous cross-protection against other serotypes lasts only a few weeks, allowing re-infection by another serotype. A number of clinical and experimental data demonstrated the implication of the immune response in the pathogenesis of severe forms of dengue, possibly through an antibody-dependant enhancement (ADE) phenomenon based on the cross-reactivity of DV antibodies [3],[4]. The molecular structure of the ectodomain of E glycoprotein has been determined [5]. It is folded in three distinct domains I, II and III. The C-terminal immunoglobulin-like domain III (EDIII) can be independently folded as a core protein through a single disulfide bond and contains major serotype-specific neutralizing epitopes [6]. On the opposite, epitopes inducing antibodies that cross-react between serotypes have been located within the domain II, which contains the fusion peptide [7]. Therefore, EDIII has emerged as an antigen of choice to develop a dengue vaccine eliciting serotype-specific rather than cross-reactive antibodies. Indeed, recent studies have demonstrated that immunization with EDIII, either encoded by a plasmid or as a recombinant protein in fusion with a bacterial carrier, elicited neutralizing antibodies to DV [8],[9],[10],[11],[12].

A preventive dengue vaccine needs to protect unexposed individuals against all four serotypes of DV. It must be tetravalent, safe for 9–12 months children and provide long-lasting protective immunity. It must be produced at low cost and scaled up at million doses. To address these challenges, we evaluated the immunogenicity of a live recombinant vector derived from pediatric measles vaccine (MV) expressing a DV antigen designed to induce neutralizing and non cross-reactive antibodies. MV vaccine is a live-attenuated negative-stranded RNA virus proven to be one of the safest, most stable, and effective human vaccines developed so far. Produced on a large scale in many countries and distributed at low cost through the Extended Program on Immunization (EPI) of WHO, this vaccine induces life-long immunity after a single injection [13],[14],[15] and boosting is effective. We previously developed a vector derived from the live-attenuated Schwarz strain of MV [16] that expressed stably different proteins from HIV and induced strong and long-term specific humoral and cellular immune responses [17],[18]. Based on this approach a program of clinical trials was initiated in collaboration with an industrial vaccine manufacturer with funding from the EC, to evaluate the safety and immunogenicity in humans of MV encoding an HIV antigen. We also demonstrated that recombinant MV could protect against flaviviruses, since MV expressing the secreted form of the E protein from West Nile virus (WNV) induced sterilizing humoral immunity against WNV in a mouse model [19].

In the present work, we evaluated the immunogenic potential of a MV vector expressing a DV1 soluble antigen composed of the EDIII fused with the ectoM. In a mouse model of MV infection this vector induced serotype-specific, virus-neutralizing antibodies against DV1. Consistent with this observation, we showed that infection of human monocyte-derived dendritic cells (DCs) resulted in up-regulation of co-stimulatory molecules as well as robust secretion of cytokines and chemokines that are identified as playing a pivotal role in establishment of anti-viral immune responses.

## Materials and Methods

### Cell culture

Vero (African green monkey kidney) cells were maintained in DMEM-Glutamax (Gibco-BRL) supplemented with 5% heat-inactivated fetal calf serum (FCS, Invitrogen, Frederick, MD). Helper 293-3-46 cells (a gift from M. A. Billeter, Zurich University) used for viral rescue [20] were grown in DMEM/10% FCS and supplemented with 1.2 mg of G418/ml. The human monocytic cell line U937 (ATCC CRL 1593, American Type Culture Collection, Rockville, Md.) was maintained in complete RPMI (Gibco-BRL) supplemented with 10% FCS (Invitrogen), sodium pyruvate, non-essential amino acids, penicillin G (100 IU/ml), and streptomycin (100 µg/ml). Clinical-grade DCs were prepared as described elsewhere [21],[22]. DCs were maintained in AIMV medium containing 500 U/mL GM-CSF (Gentaur, Brussels, Belgium) and 50 ng/mL IL-13 (Peprotech, Tebu-bio, Rocky Hill, NJ).

### Construction of pTM-MVSchw-EDIII and pTM-MVSchw-EDIII-ectoM plasmids

The plasmid pTM-MVSchw, which contains an infectious MV cDNA corresponding to the anti-genome of the Schwarz MV vaccine strain, has been described elsewhere [16]. The genomic RNA of DV-1 strain FGA/89 [23] (Genbank accession number AF 226687) was extracted from purified virions and reverse transcribed using Titan One-Step RT-PCR kit (Roche Molecular Biochemicals) according to the manufacturer's instructions. The coding sequence for PrM/E (amino acids 1-395) was cloned into pMT/Bip/V5-His A plasmid (kindly provided by Erika Navarro-Sanchez) and used as a template for further cloning. A PCR fragment encoding the EDIII (aa 295-394) from the E protein was amplified by High Fidelity Polymerase (PCR expand High Fidelity, Invitrogen) using the forward primer 1EDIII 5′-AATTAAGATCTAAAGGGATGTCATATGTGATGTG-3′ containing a *BglII* restriction site (underlined), and the reverse primer 2EDIII 5′-TTAAGCGGCCG**C** **TA**TCGCTTGAACCAGCTTAGTTTC-3′ containing a *NotI* restriction site (underlined) and a stop codon (in bold). The sequence encoding the EDIII from the E protein (aa 295-394) linked to the ectodomain of the membrane M protein (aa 1-40) by the original furin-like cleavage site RRDKR, was generated as follows. The FGA/89 EDIII (Genbank accession no. AF 226687) was amplified using the forward primer 1EDIII and the reverse primer 3EDIII 5′-CGGAACGTTTGTCTCGTCGGAACCAGCTTAGTTTCAAAGC-3′ containing the reverse complement sequence of the furin site of the DV ectoM protein (underlined) and in 3′ the reverse complement sequence of the 3′ EDIII end. The PCR product was used as primer and template to amplify by a second PCR the chimeric sequence EDIII-ectoM (Genbank accession no. CS479843) using 1EDIII primer and the reverse primer 5EDIII 5′-TTAAGCGGCCGCTATCATGGGTGTCTCAAAGCCCAAG-3′ that contains a *NotI* restriction site (underlined) and a stop codon (in bold). The cloned sequences respect the “rule of six”, which stipulates that the number of nucleotides into the MV genome must be a multiple of 6 [24]. A shuttle plasmid (pTRE2-ssCRT) containing the human calreticulin signal sequence was generated by transferring the calreticulin-derived endoplasmic reticulum targeting signal sequence from the pEGFP-RE vector (Clontech) to the pTRE2-Hyg plasmid (Clontech). The DV-1 cDNAs were introduced into pTRE2-ssCRT using *BglII/NotI* digestion. After sequencing, the 384 bp coding for the EDIII and 516 bp coding for the EDIII-ectoM antigens were inserted into *BsiWI/BssHII*-digested pTM-MVSchw-ATU2, which contains an additional transcription unit (ATU) between the phosphoprotein (P) and the matrix (M) genes of the Schwarz MV genome [16],[18],[19]. The resulting plasmids were designated as pTM-MVSchw-EDIII and pTM-MVSchw-EDIII-ectoM.

### Rescue of recombinant MV-EDIII and MV-EDIII-ectoM from the cloned cDNA

Rescue of recombinant Schwarz MV from the plasmids pTM-MVSchw-EDIII and pTM-MVSchw-EDIII-ectoM was performed as previously described [16] using the helper-cell-based rescue system described by Radecke et al [20] and modified by Parks *et al.* [25]. The titers of MV-EDIII and MV-EDIII-ectoM were determined by an endpoint limit-dilution assay on Vero cells. The TCID_50_ was calculated by use of the Kärber method.

### Production of recombinant EDIII and EDIII-ectoM proteins in Drosophila S2 cells

The EDIII and EDIII-ectoM PCR products described above were cloned into pMT/Bip/V5-His A plasmid (Invitrogen) between *BglII* and *NotI* restriction sites. The clones were validated by sequencing. *Drosophila* S2 cells (Invitrogen) were transfected by these plasmids using the Calcium Phosphate Transfection Kit (Invitrogen). Transfected cells were selected by adding 25 µg/ml blasticidin. The EDIII and EDIII-ectoM protein production was induced by adding 750 µM CuSO_4_. Cell culture supernatant was filtered on 0.2 µM filters before concentration on 10,000-MWCo Vivaspin columns (Vivasciences) eluted with PBS. Recombinant proteins were semi-quantified by Western blot using the MAb 9D12 reactive to EDIII from DV [26].

### Production of recombinant EDIII proteins in E. coli

The following DV strains from the collection of Institut Pasteur were used: strain FGA/89 French Guiana for serotype DV1 [27], Jamaica/N.1409 for DV2 [28], PaH881/88 Thailand for DV3 and 63632/76 Burma for DV4. The E. coli strain BL21(DE3) and SB medium have been described [29]. Plasmids pLB11, pLB12, pLB13, pLB14, coding respectively for EDIII from DV serotypes 1, 2, 3, 4 (residues 296-400) with an hexahistidine tag in C-term, under control of the T7 promoter and pelB signal sequence, were constructed by insertion of RT-PCR products obtained with primers specific for EDIII, in plasmid pET20b+ (Novagen) (O. Lisova et al., in preparation). The DV-EDIII-H6 recombinant proteins were produced from these plasmids in E. Coli BL21(DE3). Bacteria were grown at 24°C in SB medium with ampicillin (200 µg/mL) until A_600nm_ = 1.5 to 2.0 and then induced for 2.5 hours with 1 mM IPTG to obtain the expression of the recombinant genes. The purification of DV-EDIII-H6 proteins from bacteria's periplasmic fluid was performed by chromatography on a NiNTA resin (Qiagen, Hilden) and concentration determined by absorbance spectrometry as previously described [30]. The protein fractions were analyzed by SDS-PAGE in reducing conditions. The fractions that were homogeneous at >95%, were pooled, dialyzed against 50 mM Tris-HCl, pH 8.0, 50 mM NaCl, snap frozen in liquid nitrogen, and stored at −80°C.

### Synthetic ectoM peptide

A synthetic 40-residue long peptide corresponding to the ectoM sequence from FGA/89 strain (SVALAPHVGLGLETRTETWMSSEGAWKQIQKV ETWALRHP) was synthesized with a purity of at least 80% (Genecust, France).

### Western blot assays

Protein lysates from Vero cells or U937 cells infected with recombinant viruses were fractionated by SDS-PAGE gel electrophoresis and transferred to cellulose membranes (Amersham Pharmacia Biotech). DV1 EDIII (5 ng) produced in drosophila cells was loaded as a positive control. The blots were probed with a murine monoclonal antibody mAb4E11 directed against the E EDIII of DENV1 (Hybridoma cells producing 4E11 raised against the envelope protein of DV1 were kindly provided by Dr Morens [30]). A goat anti-mouse immunoglobulin G (IgG)-horseradish peroxidase (HRP) conjugate (Amersham) was used as a secondary antibody. Peroxidase activity was visualized with an enhanced chemiluminescence detection kit (Pierce).

### Apoptosis analysis

Cells were recovered by pipetting, centrifuged for 3 min at 1200 rpm, washed once in PBS and resuspended in FITC-labeled annexinV/propidium iodide (PI) according to the manufacturer's instructions (Becton Dickinson, Apoptosis Detection kit). Labeled cells were analyzed by flow cytometry using a FacsCalibur (BD Biosciences, San Diego, CA), with CellQuest software (Becton Dickinson, Lincoln Park, NJ). The percentage of apoptotic cells was determined as the percentage of AnnexinV positive and propidium iodide negative cells.

### Immunofluorescence

Immunofluorescence staining was performed on infected cells, as described elsewhere [31]. Cells were probed with mouse anti DV-1 EDIII 4E11 antibody, mouse anti DV-1 HyperImmune Ascitic Fluid [32] or rabbit anti human MHC-II dimer (kindly provided by Neefjed J.) antibodies. Cy3-conjugated goat anti mouse IgG antibody Cy3 conjugated (Jackson Immunoresearch laboratories), FITC-conjugated goat anti-mouse IgG antibody (Chemicon), or and FITC-conjugated goat anti-rabbit IgG antibody (Amersham Pharmacia Biotech) were used as secondary antibodies respectively.

### DC phenotypic analysis

Cell-surface staining was performed at 4°C for 30 minutes using anti-CD86-PE (BD Pharmingen), CD83-APC (BD Pharmingen), CD80-PE-Cy5 (Immunotech, Marseille) in 1% BSA and 3% human serum-PBS. Isotype-matched mAbs were used as negative controls. Labeled cells were analyzed by flow cytometry using a FacsCalibur (BD), with FlowJo software (Tree Star, Ashland, OR).

### Detection of cytokines in DC supernatants

Supernatants were harvested after 16 h or 24 h of DC incubation with MV-EDIII or MV-EDIII-ectoM at an MOI of 1. Aliquots of 200–300 µl were stored at −80°C. Production of cytokines/chemokines was analyzed in 50 µL supernatant with a human cytokine 25-plex antibody bead kit (Biosource, CA, WA, cat. LHC0009) which measures IL-1α, IL-1Ra, IL-2, IL-2R, IL-4, IL-5, IL-6, IL-7, IL-8, IL-10, IL-12p40/70, IL-13, IL-15, IL-17, TNF-α, IFN-α, IFN-β, GM-CSF, MIP-1α, MIP-1β, IP-10, MIG, Eotaxin, RANTES and MCP-1 by using a Luminex 100 instrument (Luminex Corp., Austin, TX, USA).

### Mice experiments

CD46-IFNAR mice susceptible to MV infection were produced as previously described [16]. Mice were housed under specific pathogen-free conditions at the Pasteur Institute animal facility. Six-week-old CD46-IFNAR mice were inoculated intraperitoneally (i.p.) with 10^4^ or 10^5^ TCID_50_ of recombinant MV. Boosting was performed using 10 µg of recombinant EDIII-ectoM protein in Alugel adjuvant. To detect the anamnestic response generated by immunization, immunized mice were i.p. inoculated with 10^7^ FFU of live FGA/NA d1d variant of DV1 (Genebank accession number AF 226686). This strain was previously generated by adaptation of a clinical isolate to growth in newborn mouse brain [23]. All experiments were approved and conducted in accordance with the guidelines of the Office of Laboratory Animal Care at Pasteur Institute.

### ELISA for humoral responses

To evaluate the specific antibody responses, mice were bled via the periorbital route at different time after inoculation. Sera were heat inactivated at 56°C for 30 min and the presence of anti-MV antibodies was detected by ELISA (Trinity Biotech). HRP-conjugated anti-mouse immunoglobulin (Jackson Immuno Research) was used as secondary antibody. Anti-DV antibodies were detected by ELISA using 96-wells plates coated with either highly purified FGA/89 DV1 particles, recombinant EDIII proteins from DV1, DV2, DV3, DV4 produced in *E. Coli.* or synthetic ectoM peptide. HRP-conjugated anti-mouse immunoglobulin was used as secondary antibody. The endpoint titers of pooled sera were calculated as the reciprocal of the last dilution giving twice the absorbance of sera from MV inoculated mice that served as negative controls.

### Focus reduction neutralization test

Anti-DV neutralizing antibodies were detected by a focus reduction neutralization test (FRNT) on Vero cells previously described [19] using 50 FFU of Vero-adapted DV1 Hawaï (WHO reference strain, Genbank accession no. AF226687), DV2 Jamaica (Genbank accession no. M20558), DV3 H97 (WHO reference strain, Genbank accession no. M93130) or DV4 63632. The endpoint titer was calculated as the highest serum dilution tested that reduced the number of FFU by at least 50% (FRNT_50_) or 75% (FRNT_75_). For the neutralization tests in presence of recombinant EDIII or synthetic peptides ectoM peptides serum samples were pre-incubated (in 50 µl medium) with 5 µg, 500 ng or 50 ng of recombinant EDIII (produced in Drosophila S2 cells) or synthetic ectoM peptide before performing FRNT.

## Results

### Recombinant viruses MV-EDIII and MV-EDIII-ectoM express the EDIII antigen, lead to its secretion, and replicate efficiently

**Figure 1:**
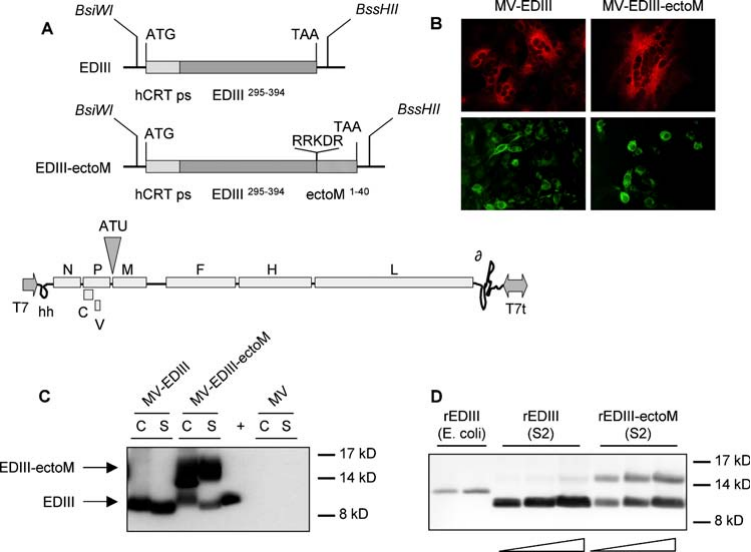
Expression of DV1 EDIII and EDIII-ectoM by recombinant MV vector. (A) Schematic representation of DV1 EDIII and EDIII-ectoM constructs and of recombinant MV vector. The human calreticulin peptide signal sequence (hCRT ps), the DV1 envelope E domain III (EDIII) and the M ectodomain (ectoM) are indicated. Original furin-like cleavage site RRDKR and amino acid positions are indicated. The EDIII and EDIII-ectoM sequences were cloned into the ATU position 1 of MV vector using *BsiWI/BssHII* sites. The MV genes are indicated as follows: nucleoprotein (N), phosphoprotein and V/C accessory proteins (PVC), matrix (M) fusion (F), hemagglutinin (H) and polymerase (L). T7 RNA polymerase promoter (T7), T7 RNA polymerase terminator (T7t), hepatitis delta virus ribozyme (∂), hammerhead ribozyme (hh). (B) Detection of DV1 EDIII in Vero cells infected for 24h with MV-EDIII or MV-EDIII-ectoM. EDIII was detected in two different experiments using either anti-EDIII 4E11 antibody (red label) or anti-DV1-HMAF (green label). (C) DV1 EDIII expression in cell lysates (C) and supernatants (S) of Vero cells infected by MV-EDIII and MV-EDIII-ectoM analyzed by western blot (cell lysates are 20 times more concentrated than supernatants). (+ ) positive control (5 ng of DV1 EDIII produced in drosophila S2 cells). DV1 EDIII was probed with the 4E11 mouse monoclonal anti-DV1 EDIII. (D) Western blot analysis of recombinant rEDIII protein produced in E.coli (100 ng) and recombinant rEDIII or rEDIII-ectoM proteins produced in drosophila S2 cells supernatants (0.5, 1 and 2 µg/well). DV1 EDIII was probed with 4E11 mouse monoclonal anti-DV1 EDIII.

We cloned from DV1 viral RNA the sequence encoding the EDIII (E_295-394_) fused in C-term to the ectoM (M_1-40_), using as a linker the original prM/M furin-like cleavage site RRDKR. This combined dengue antigen was cloned downstream the cellular calreticulin (ss-CRT) signal peptide sequence in order to allow the disulfide bond formation and therefore the correct folding of EDIII and to address the antigen in the secretion pathway. As a control of proper folding and effective secretion of EDIII, we also generated a similar construct without ectoM. The resulting ss-CRT-EDIII-ectoM and ss-CRT-EDIII constructs were inserted into MV vector (pTM-MVSchw plasmid), which contains an infectious MV cDNA corresponding to the anti-genome of the Schwarz MV vaccine strain [16] (Figure 1A). The recombinant measles viruses MV-EDIII-ectoM and MV-EDIII were rescued by transfecting the pTM-MVSchw-EDIII-ectoM and pTM-MVSchw-EDIII plasmids into helper cells and propagation on Vero cells, as previously described [16].

We analyzed the expression of DV antigens by recombinant MV in infected Vero cells by immunofluorescence using a monoclonal neutralizing anti-DV1 EDIII antibody (4E11, [33]) and anti-DV1 Hyper Immune Ascitic Fluid (HMAF) (Figure 1B). In both cases, the antigens were clearly detected indicating that the EDIII was expressed and that the epitope of 4E11 neutralizing antibody was present and accessible. The presence of EDIII in lysates and supernatants of infected Vero cells was further confirmed by Western blot using 4E11 antibody (Figure 1C). DV1 recombinant EDIII polypeptides (rEDIII and rEDIII-ectoM) secreted from stable S2 cell lines were used as positive controls (Figure 1D). DV1 EDIII antigen was detected both in lysates and supernatants from cells infected by MV-EDIII-ectoM and MV-EDIII vectors. The EDIII was clearly detected in unconcentrated supernatant, despite that the supernatant volume was 100 times larger than the lysate volume. Thus, secretion was efficient. The intracellular EDIII shows a higher molecular weight on the western blot than EDIII secreted in the supernatants, because of the presence of the peptide signal, which was cleaved during secretion. Similarly, the positive control (rEDIII from S2 cells) has a higher molecular weight because it contains a poly-histidine tag. Cells infected by MV-EDIII-ectoM produced cleaved EDIII and uncleaved EDIII-ectoM antigens both in cell lysates and medium, indicating that the furin-like cleavage site was accessible. Taken together, these data show that MV-EDIII-ectoM vector is able to produce secreted forms of EDIII, EDIII-ectoM, and by assumption, ectoM (not detected because of the lack of specific antibodies to ectoM).

**Figure 2:**
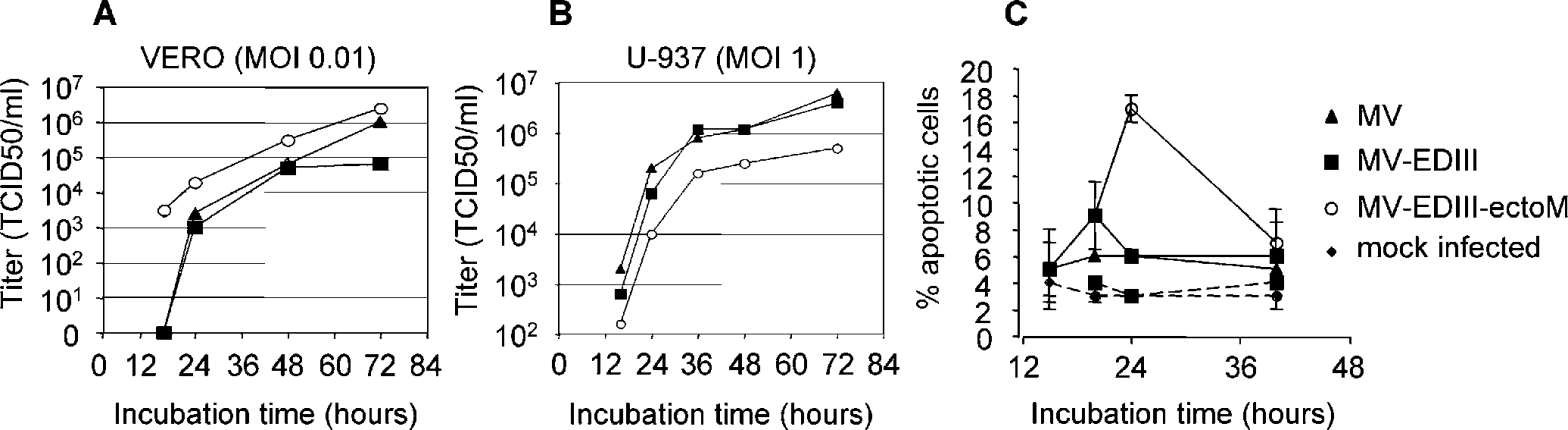
In vitro replication and cytopathic effects induced by recombinant MV-DV. (A, B) Growth kinetics of recombinant MV-EDIII and MV-EDIII-ectoM viruses compared with standard MV on Vero and U-937 cells (MOI 0.01 and 1, respectively). Cell-associated virus titers are indicated in TCID_50_. (C) Apoptosis of DCs infected by recombinant viruses (MOI 10). MV (triangles), MV-EDIII (squares), MV-EDIII-ectoM (open circles), mock infected (diamonds). UV inactivated viruses (same symbols with dotted lines). The percentage of apoptotic cells represents the percentage of Annexin-V positive and propidium iodide negative cells (determined using a flow cytometer). Values are means ± SE from two independent experiments, each performed in duplicate.

We analyzed the replication of MV-EDIII-ectoM and MV-EDIII viruses on Vero cells using the same MOI (0.01) than for MV production. The growth kinetics of both recombinant viruses were similar to that of control MV and the final titer was slightly higher for MV-EDIII-ectoM (Figure 2A). We then investigated whether the presence of ectoM in MV-EDIII-ectoM virus could increase apoptosis of infected cells [2]. Since human monocytes constitute a determinant target of MV infection for initiation of immune responses, we addressed this question by infecting human monocytic cells (U937 leukemic monocyte lymphoma cell line). Growth kinetics of MV, MV-EDIII and MV-EDIII-ectoM in U937 cells (MOI 1) show that these cells are permissive to MV infection and that MV-EDIII-40 growth was slightly delayed as compared to MV and MV-EDIII (Figure 2B). We quantified apoptotic cells (annexin V positive/propidium iodide negative cells) after infection at different MOI 0.1, 1 and 10. While we did not observe apoptosis up to 42 hours post-infection when using MOIs of 0.1 or 1 with the 3 viruses, increasing the MOI to 10 with MV-EDIII-ectoM virus eventually induced apoptosis in 15% of cells (Figure 2C). Apoptosis was related to the activation of caspase 3 pathway (not shown) and was dependent on virus replication, since UV inactivated virus did not trigger apoptosis.

### MV-EDIII-ectoM induces DV1 neutralizing antibodies in mice

We examined the ability of MV-EDIII-ectoM recombinant virus to raise specific anti-DV1 neutralizing antibodies in genetically modified mice susceptible to MV infection [34]. These mice express CD46, the human receptor for vaccine MV strains, and lack the INF-α/β receptor (IFNAR) [16],[18],[35],[36]. They have previously been used as a model to evaluate the immunogenicity of recombinant MV [16],[17],[18],[19],[36]. Six-week-old CD46-IFNAR mice received two intraperitoneal (ip) injections within one month of either 10^4^ or 10^5^ TCID_50_ of MV-EDIII-ectoM. As a control, CD46-IFNAR mice were immunized with MV-EDIII and empty MV vector. Specific antibody responses were analyzed by ELISA one month after the second injection (Table 1). All immunized mice raised antibodies to MV at similar titers. Specific anti-DV1 and anti-rEDIII antibodies were mounted in mice immunized with MV-EDIII-ectoM (titers 3,000 and 10,000 respectively). Surprisingly, no anti-DV antibodies were detected in sera of mice immunized with MV-EDIII. An ELISA test using ectoM as coated viral antigen showed that immune sera had no detectable levels of anti-M antibodies. We evaluated the anti-DV1 neutralizing activity of immune sera by using a focus reduction neutralization test (FRNT) that allows to determine the highest serum dilutions able to reduce by at least 50% or 75% the number of DV1 focus forming units (FFU) on Vero cells. Again, whereas immunization with MV or MV-EDIII did not induce neutralizing antibodies to DV1, immunization by MV-EDIII-ectoM raised FRNT_50_ titers to 320 and FRNT_75_ titers to 40 (Table 1). Altogether, these data show that EDIII-ectoM is able to elicit humoral immunity to DV.

**Table 1:** Antibody response of CD46-IFNAR mice to immunization with MV-EDIII or MV-EDIII-ectoM.

Injections	MV Ab titer^c^	DV1 Ab titer^c^	rEDIII Ab titer^c^	ectoM Ab titer^c^	Anti-DV1 FRNT50^d^	Anti-DV1 FRNT75^e^
MV^a^	100,000	<100	<100	<100	<10	<10
DV1^b^	10,000	3,000	1,000	<100	320	160
MV-DIII^a^	100,000	<100	<100	<100	<10	<10
DV1 b	10,000	3,000	1,000	<100	320	160
MV-EDIII-ectoM^a^	100,000	3,000	10,000	<100	320	40
DV1 b	10,000	30,000	300,000	<100	8000	2560–5120

a CD46-IFNAR mice were inoculated intraperitoneally (i.p.) twice with 10^5^ TCID_50_ of MV, MV-EDIII or MV-EDIII-ectoM at one month of interval (6 mice/group). ^b^Two months after the last immunization, mice were inoculated with 10^7^ FFU of DV1 FGA/NA d1d and sera were analyzed 3 weeks after. ^c^Antibody titers were determined by ELISA on pooled heat-inactivated sera collected one month after immunization and DV inoculation. ^d^FRNT_50_ represents the highest serum dilution that reduced the number of DV focus-forming units (FFU) on Vero cells by at least 50% and ^e^FRNT_75_ by at least 75%.

### Anamnestic response generated by immunization with MV-EDIII-ectoM is strongly boosted by intraperitoneal inoculation of live DV

Although CD46-IFNAR mice used in this study did not allow to evaluate the protection conferred by immunization, we tested the ability of live DV1 peripheral inoculation to stimulate long-term anamnestic humoral response against MV-EDIII-ectoM. To assess first the susceptibility of CD46-IFNAR mice to DV1 replication, we inoculated mice intraperitoneally with 10^7^ FFU of DV1 strain FGA/NA d1d. No symptoms or mortality were observed and virus replication could not be detected in mice serum by direct plaque assay on mosquito cells (not shown). However, all groups of mice seroconverted after live DV1 inoculation (Table 1). Interestingly, the anamnestic memory induced by immunization with recombinant MV-EDIII-ectoM was remarkably boosted by live DV1 inoculation, whereas animals immunized with MV-EDIII or empty MV did not show any boost (Table 1). Both ELISA and FRNT neutralizing titers against DV1 were strongly increased in mice immunized with MV-EDIII-ectoM (30–100 times increase), showing evidence of an efficient anamnestic response.

To test the longevity of this memory, another group of mice was primed with two injections of MV-EDIII-ectoM vector (Table 2). Six months later, they were boosted by injecting 5 µg of adjuvanted rEDIII-ectoM protein purified from supernatants of transfected drosophila S2 cells. Protein boost increased the neutralizing titer from 10 to 200. However, the titer decreased rapidly to 40, indicating a transient boost. As a control, mice inoculated only with the recombinant protein remained negative even after three injections (not shown). At 9 months post priming, mice were i.p. inoculated with 10^7^ FFU of live FGA/NA d1d DV1. One month after DV1 inoculation, we again observed a 100 times increase in the level of antibodies to DV1 and to EDIII as well as DV1 neutralizing titers (Table 2). This experiment shows that neutralizing antibodies to DV EDIII induced by MV-EDIII-ectoM are efficiently boosted upon live DV exposure 9 months after priming, and demonstrates the induction of a durable B-cell memory. However, the protection against DV infection needs to be evaluated in a more appropriate non-human primate model.

**Table 2:** Long-term antibody response of CD46-IFNAR mice to immunization with MV-EDIII-ectoM.

Injections^a^ (time, months)	Time of sera collection (months)	MV Ab Titer^b^	DV-1 Ab Titer^b^	DV-1 rEDIII Titer^b^	Anti-DV1 FRNT50^c^	Anti-DV1 FRNT75^d^
MV-EDIII-ectoM (0)	1	15,000	<100	≤ 100	nd	nd
MV-EDIII-ectoM (1)	2	40,000	1,600	400	40	10
	3	30,000	1,000	600	40	10
	5	20,000	500	100	40	10
rEDIII-ectoM (6)	7	20,000	20,000	100,000	1600	200
	9	10,000	2,000	10,000	320	40
DV1 inoculation (9)	10	10,000	200,000	800,000	32,000	4,000

a CD46-IFNAR mice were inoculated intraperitoneally (i.p.) twice with 10^4^ TCID_50_ of MV-EDIII-ectoM at one month of interval. At 6 months, mice were boosted with 10 µg of recombinant DV1 rEDIII-ectoM protein (from S2 cells) in Alugel adjuvant. At 9 months, mice were inoculated with 10^7^ FFU of DV-1 FGA/NA d1d. ^b^Determined by ELISA on pooled heat-inactivated sera collected one month after immunizations or DV infection. ^c^FRNT_50_ represents the highest serum dilution that reduced the number of DV focus-forming units (FFU) on Vero cells by at least 50% and ^d^FRNT_75_ by at least 75%.

### Anti-DV1 neutralizing antibodies raised by MV-EDIII-ectoM immunization are specific of DV1-EDIII and do not cross-react with other serotypes

**Figure 3:**
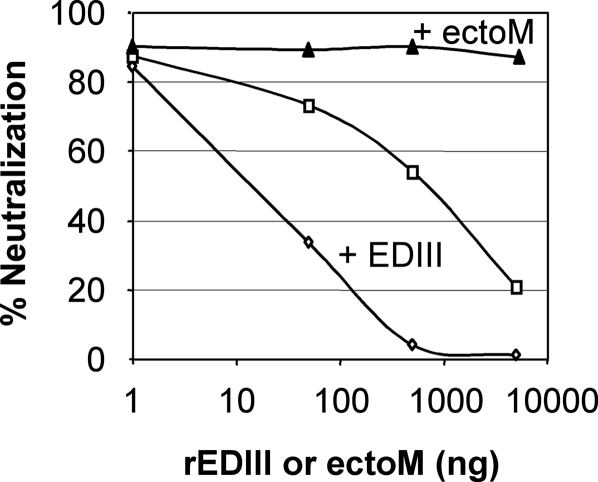
Neutralization tests in presence of recombinant rEDIII protein or synthetic ectoM peptide. The neutralization activity of immune sera collected before DV exposure (diamonds and triangles, 1/100 dilution) and post DV exposure (open squares 1/1000 dilution) was tested in presence of increasing doses of recombinant EDIII (produced in drosophila cells) or ectoM synthetic peptide. Pools of sera were preincubated with rEDIII or ectoM for 1h at 37°C before FRNT_75_ titration on Vero cells (50 FFU DV1 FGA89 for 2h at 37°C).

To assess the DV serotype specificity of antibodies induced, we tested mice sera from experiment presented in table 2 by ELISA against rEDIII proteins from DV1, 2, 3 and 4, respectively. We also evaluated the anti-DV1, anti-DV2, anti-DV3 and anti-DV4 neutralizing activity of immune sera by FRNT_50_. Antibodies induced by recombinant MV-EDIII-ectoM were specific to DV1 and did not cross-react with the EDIII from the other serotypes of DV (Table 3). This confirms that EDIII antigenic surface is serotype specific. To determine whether the antiviral neutralizing activity induced by MV-EDIII-ectoM was specifically directed against the EDIII, even after live DV1 inoculation, we performed neutralization tests in presence of increasing concentrations of either rEDIII protein produced in drosophila cells or synthetic ectoM peptide (Figure 3). Increasing concentrations of rEDIII protein strongly reduced the antiviral activity of sera collected before and after live DV1 inoculation, whereas ectoM peptide was ineffective. This experiment demonstrates that the neutralizing antibodies induced by immunization were specifically directed against EDIII epitopes essential for virus infectivity. Moreover, the antiviral activity of sera collected after live DV1 inoculation was also completely inhibited by rEDIII protein, indicating that live DV1 inoculation increased the level of antibodies already induced by immunization, but did not raise new antibodies directed to other neutralizing epitopes than EDIII.

**Table 3:** Serotype specificity of antibodies induced in CD46-IFNAR mice after immunization with recombinant vectors.

	DV1		DV2		DV3		DV4	
	Ab titer^a^	FRNT50^b^	Ab titer^a^	FRNT50^b^	Ab titer^a^	FRNT50^b^	Ab titer^a^	FRNT50^b^
MV	3,000	160	<100	nd	<100	nd	<100	nd
MV-EDIII	3,000	160	<100	40	<100	40	<100	40
MV-EDIII-ectoM	300,000	10240	500	40	<100	20	<100	10

a Determined by ELISA using plates coated with rEDIII from DV1, 2, 3 or 4 produced in E. coli (pooled heat-inactivated post-DV inoculation sera from experiment presented in Table 2).

b FRNT50 is the highest serum dilution that reduced the number of DV focus-forming units (FFU) on Vero cells by at least 50%.

Altogether, these experiments demonstrate that recombinant MV-EDIII-ectoM virus induced specific antibodies to DV1 EDIII that did not cross-react with other DV serotypes and that neutralized specifically DV1 infection. The EDIII alone expressed by recombinant MV was poorly immunogenic, although it was expressed and secreted at levels similar to EDIII-ectoM. The presence of the ectoM was determinant to its immunogenicity, raising the question of the mechanism of this effect. Does the presence of ectoM in C-term of the EDIII sequence improve the conformation of EDIII to make it biologically active, or adjuvant its immunogenicity through an indirect mechanism? The EDIII secreted from cells infected by MV-EDIII was recognized by neutralizing antibody 4E11, which binds to the active receptor-binding form of EDIII [33] The same EDIII sequence secreted by drosophila cells was able to efficiently compete with the neutralizing activity of antibodies induced by the MV-EDIII-ectoM virus, thus suggesting that EDIII was biologically functional, at least able to present neutralizing epitopes. To address the question of adjuvantation, we compared *in vitro* the effect of MV-EDIII-ectoM and MV-EDIII infection on human immature monocyte-derived DCs (MDDCs) in terms of activation/maturation and cytokine/chemokine secretion.

### MV-EDIII-ectoM replication enhances maturation of DCs and cytokine/chemokine secretion

**Figure 4:**
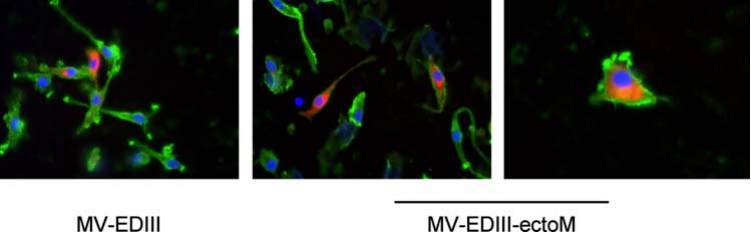
Detection of DV1 EDIII in human DCs incubated for 17h with MV-EDIII or MV-EDIII-ectoM (MOI 1). EDIII was detected using anti-EDIII 4E11 antibody (red label). Anti-human MHC-II dimer primary antibody was used as a DC marker (green label) (kindly provided by Neefjed J.). Cells nuclei were labeled with DAPI.

**Figure 5:**
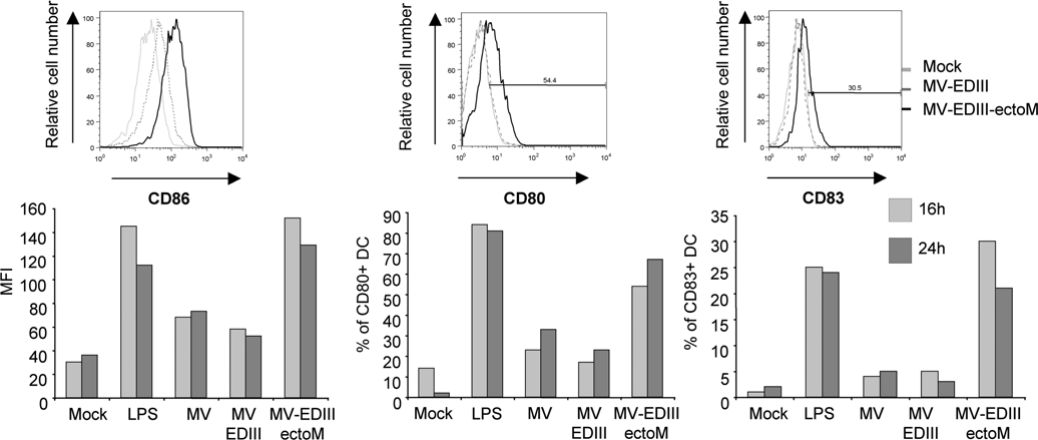
Expression of CD86, CD80, CD83 cell surface molecules on human DCs incubated in presence of MV-EDIII and MV-EDIII-ectoM (MOI of 1), or mock treated. The expression of cell surface markers was analyzed after 15h and 24h incubation by flow cytometry. Data shown are representative of three individual experiments. Thin light-grey line represent staining of mock-treated DC, dashed grey lines represent staining of MV-EDIII-infected DCs, and thick black line represent staining of MV-EDIII-ectoM-infected DCs.

**Figure 6:**
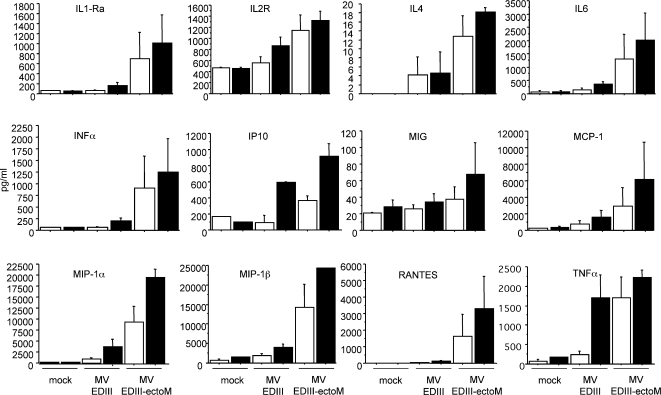
Cytokine and chemokine secretion by human DC incubated in presence of MV-EDIII and MV-EDIII-ectoM (MOI 1). The supernatants harvested at 16h (white bars) and 24h (black bars) after infection were analyzed with a multiplex assay to measure the concentration of cytokines and chemokines. The values are means ± SE from four independent experiments.

Upon virus infection, immature dendritic cells (DCs) undergo maturation, and transport the virus to regional lymph nodes, where viral antigens are presented to lymphocytes to initiate immune response [37]. DCs are permissive to MV infection [38] leading to the up-regulation of co-stimulatory molecules [39],[40],[41]. To evaluate *in vitro* the effect of recombinant MV-DV on DC activation, we cultivated human immature monocyte-derived DCs (MDDCs) in presence of MV-EDIII-ectoM or MV-EDIII viruses. After 17 hours of infection, DCs expressed the DV1 EDIII as detected by immunofluorescence (Figure 4). We then analyzed the kinetics and levels of expression of costimulatory molecules on the surface of DCs. The three viruses MV-EDIII-ectoM, MV-EDIII and MV promoted the up-regulation of CD86, CD83 and CD80 molecules as compared to mock-treated DCs (Figure 5). Remarkably, MV-EDIII-ectoM up-regulated these molecules more extensively and at earlier time points than MV-EDIII and MV. Viral replication and *de novo* synthesis of EDIII-ectoM were required since UV-inactivated recombinant viruses or synthetic ectoM peptide had no effect (not shown). To determine if the increased capacity of MV-EDIII-ectoM to activate DCs correlated with phenotypic functional changes, we analyzed the secretion of 23 cytokines/chemokines in the supernatant of DCs cultivated for 16h and 24h in presence of MV-EDIII-ectoM or MV-EDIII. As a control, DCs were mock-infected or cultivated in presence of LPS. We found that infection with MV-EDIII or MV-EDIII-ectoM induced a panel of cytokines and chemokines consistent with other reports on DC infection by MV (Table 4) [42]. Remarkably, some cytokines and chemokines were significantly enhanced and/or induced at earlier time points by MV-EDIII-ectoM as compared to MV-EDIII (Table 4, Figure 6). Among them, IFN-α (1000 pg/ml at 16 h), IL1RA (750 pg/m), IL4 (13 pg/m) and the proinflammatory cytokines IL-6 (1250 pg/m) and TNF-α (1700 pg/m) were induced much more rapidly after MV-EDIII-ectoM infection and at levels 8–10 times higher than after MV-EDIII infection (Figure 6). Such a strong enhancement in production of these cytokines is expected to accelerate the establishment of immune response and to favor humoral immunity. Similarly, remarkable levels of MIP-1α chemokine were induced by MV-EDIII-ectoM at early time points (9,000–20,000 pg/ml). A similar robust and early secretion was observed for RANTES, MIP-1β and MCP-1α. The production of these chemokines by maturing DCs may promote the recruitment of other antigen presenting cells (APC) such as immature DCs to enhance and sustain antigen sampling, and polarization of the immune response [43],[44],[45]. Thus, adding the ectoM protein in fusion with EDIII resulted in a stronger and faster maturation of human DCs and activated the secretion of higher levels of inflammatory and antiviral cytokines as well as chemokines determinant for the establishment of specific immune responses.

**Table 4:** Cytokine and chemokine secretion induced in human DCs by MV-EDIII and MV-EDIII-ectoM.

	MV-EDIII	MV-EDIII-ectoM
IL-1β	-	-
IL2	-	-
IL5	-	-
IL10	-	-
INFγ	-	-
Eotaxin	-	-
IL7	+	+
IL8	++	++
IL12p40-70	+	+
IL15	+	+
IL17	+	+
IL1-RA	+	+++
IL2R	+	++
IL4	+	+++
IL6	+	+++
TNFα	+	++
INFα	+	+++
MCP-1	+	+++
MIG	+	++
MIP-1α	+	+++
MIP-1β	+	+++
IP10	+	++
RANTES	+	+++

Cytokine/chemokine production determined by multiplex assay in supernatants of human DCs incubated in presence of MV-EDIII or MV-EDIII-ectoM (16h incubation). Results are from four independent experiments performed with four different donors. Production levels are indicated as :

(-) basal production by mock-treated DCs

(+) less than twofold increase compared to mock-treated DCs

(++) more than threefold increase compared to mock-treated DCs

(+++) more than fourfold increase compared to mock-treated DCs

## Discussion

The objective of this study was to evaluate the immunogenicity of a dengue vaccine candidate based on a pediatric measles vaccine expressing a minimal dengue antigen. This strategy provides a recombinant vaccine that might protect children simultaneously from measles and dengue and that might be affordable to populations through the EPI program in the regions affected both by dengue and measles infections. An efficient pediatric dengue vaccine is supposed to elicit durable protective humoral immune responses against all four dengue serotypes without risk of ADE [46]. Regarding this objective, we assembled covalently the antigenic domain III from the DV1 envelope E glycoprotein and the pro-apoptotic ectodomain of DV-1 M protein to generate a dengue combined antigen, EDIII-ectoM. In the fusion construct, the N-terminal calreticulin peptide signal sequence directs EDIII-ectoM to the secretory pathway. The furin-dependent cleavage site of prM/M which links ectoM to EDIII allows the processing of the antigen by specific proteases throughout the Golgi apparatus. Expressed by recombinant MV vector, the EDIII-ectoM antigen induced in mice susceptible to MV specific antibodies to DV1 EDIII that did not cross-react with other DV serotypes and that neutralized DV1 infection in vitro. Immunization primed a long-term memory that was vigorously boosted when animals were inoculated with live DV.

Although DV disease pathogenesis and protection mechanisms are not fully clarified, disease severity is correlated with viremia levels and neutralizing antibody is generally used as a marker of vaccine effectiveness [47]. Experimental mouse models of DV infection have been reported showing that adult AG129 mice, which are deficient for IFN α/β/γ receptors develop a dose-dependant transient viremia after peripheral injection of unadapted or mouse-adapted DV, whereas A129-IFNAR mice, which are deficient only for IFN-α/β receptor are less sensitive to DV infection [48],[49],[50]. However, AG129 mice are not sensitive to MV infection and the prototype DV1 Hawaï strain did not replicate in these mice [50]. Suckling mice develop lethal encephalitis after DV intracerebral inoculation, but in our study mice were 3–4 month-old after two MV immunizations and intracranial inoculation could not be performed. Moreover, this model is far from the human situation since DV does not infect the nervous system, nor lead to encephalitis in humans. The CD46-IFNAR mouse model sensitive to MV infection that we used did not allow documenting in vivo protection from DV replication. Therefore, to demonstrate the induction of anamnestic neutralizing antibody response upon live DV exposure, mice were peripherally inoculated with DV a long time after immunization. These experiments showed that neutralizing antibodies induced by immunization with MV-EDIII-ectoM were strongly boosted by live DV inoculation, thus suggesting a protective capacity. Indeed, the available vaccines against yellow fever, Japanese encephalitis and tick-borne encephalitis viruses have proven that anamnestic neutralizing antibodies play an essential role for protection against flaviviral infections [47].

The EDIII without ectoM was poorly immunogenic in the context of MV expression. It appeared, therefore, that the 40-residue long ectodomain of M plays a critical role in its immunogenicity. DV EDIII has been previously shown to be immunogenic in the form of recombinant chimeric proteins [8],[9],[10],[12] or expressed from a plasmid [51] or from adenovirus vector [52],[53]. To determine whether the EDIII sequence inserted into MV vector was able to present neutralizing epitopes, we produced in E.coli recombinant EDIII proteins from DV1, 2, 3 and 4 corresponding to the same sequence and we coated plates with these proteins. Tested by ELISA on these plates, a neutralizing HMAF specific to DV1 recognized specifically the DV1-EDIII, but not the other serotypes (data not shown), indicating its specificity. This DV-1 HMAF recognized also specifically by immunofluorescence the DV1 EDIII expressed in cells infected by MV-EDIII, indicating the capacity of EDIII to expose serotype-specific epitopes. The neutralizing monoclonal antibody 4E11 recognized also the EDIII expressed by MV-EDIII infected cells, indicating that the epitope specific of this antibody is accessible within the EDIII expressed by MV. This epitope has been mapped and shown to be exposed on the native form of EDIII [33]. Furthermore, we expressed the same EDIII sequence as a secreted protein by drosophila cells and showed that it was able to efficiently compete with the neutralizing activity of antibodies induced by the MV-EDIII-ectoM virus. Altogether, these observations suggest that EDIII expressed by MV was able to present a conformationally active neutralizing epitope. Recent studies evaluating the immunogenicity of West-Nile virus (WNV) EDIII showed that a high amount of EDIII was necessary to induce neutralizing antibodies, while EDIII fused to TLR ligands was immunogenic and conferred protection at lower doses [54],[55]. Therefore, the low immunogenicity of DV EDIII in our hands might be due to the lower amount of antigen expressed by recombinant MV as compared to the high protein or DNA doses administered by others. To increase the level of expression by MV, EDIII can be cloned upstream the N gene, as MV genes are expressed as a gradient from the 3′ to the 5′ end of the genome.

This small ectoM protein, which is highly conserved among the four serotypes of DV, has pro-apoptotic properties [2]. High titers of MV-EDIII-ectoM induced apoptosis of infected U937 monocyte-like cells that was not observed at standard titers. This critical point in terms of safety needs to be evaluated further in the development of this vaccine candidate. Indeed, recombinant MV vector has to keep the high safety level of standard MV vaccine. However, this property might be determinant to the immunogenicity of EDIII because apoptotic infected cells express Toll-like receptor (TLR) ligands that increase the cross-presentation of viral epitopes by antigen presenting cells [56],[57]. Indeed, we observed that ectoM, in the context of MV replication, increased human DCs maturation and triggered the release of cytokines and chemokines determinant for the establishment of specific adaptive immunity. Therefore, its capacity to adjuvant EDIII might be still more efficient in humans than in CD46-IFNAR mice. Further studies are needed to address the mechanism of action at the molecular level.

In conclusion, we have produced a minimal antigen from DV1 able to induce long-term specific neutralizing antibodies to DV1 with no cross-reactivity with other serotypes. We have shown that the remarkable adjuvant capacity of ectoM to EDIII immunogenicity was correlated to its capacity to mature primary DCs and to activate the secretion of a panel of proinflammatory and antiviral cytokines, as well as numerous chemokines determinant for the establishment of specific adaptive immunity. The immunogenicity of this antigen was demonstrated through its expression by a recombinant MV vector, thus making the proof-of-concept of this strategy for dengue vaccine development. Using MV as a vaccination vector presents a number of advantages : vaccination against measles is mandatory, vaccine strains are genetically stable, MV does not recombine or integrate genetic material, and vaccine does not persist or diffuse. MV-specific CD8 T cells and IgG are detected in vaccinees up to 25–34 years after a single MV vaccination [14] and boosting increases this memory [15]. Using MV as a recombinant vaccine to immunize simultaneously against measles and dengue might be particularly attractive in areas where both diseases threaten children every year, such as Africa and South America. Taking advantage of the capacity of MV vector to express large amounts of heterologous genetic material very stably [58], we generated tetravalent dengue antigenic constructs inserted into single MV vectors that are currently characterized. Such a strategy should avoid the stability and interference problems encountered with tetravalent formulation of four attenuated viruses, as well as the reactogenicity problems [59]. These new candidates will be evaluated in a much more appropriate non-human primate model.

## Supporting Information

Alternative Language Abstract S1 Translation of abstract into Spanish
(0.05 MB DOC)

Alternative Language Abstract S2 Translation of abstract into French
(0.02 MB DOC)
