# Supplementary material for: Pediatric Measles Vaccine Expressing a Dengue Antigen Induces Durable Serotype-specific Neutralizing Antibodies to Dengue Virus
Source: PLoS Negl Trop Dis. 2007 Dec 12;1(3):e96. doi: 10.1371/journal.pntd.0000096 (PMC2154386; doi:10.1371/journal.pntd.0000096)
Supplement: Alternative Language Abstract S1 — Translation of abstract into Spanish (0.05 MB DOC) [file pntd.0000096.s001.doc]

# RESUMEN

(Translation of the abstract into Spanish by authot Samantha Brandler)

La fiebre del dengue es una enfermedad emergente que amenaza un tercio de la población mundial. A pesar de varias décadas de esfuerzos, no existe vacuna contra el dengue. Con el objetivo de desarrollar una vacuna económica que podría ser utilizada en poblaciones jóvenes en zonas tropicales, en este estudio evaluamos una nueva estrategia basada en la expresión de un antígeno de dengue por un vector derivado de la vacuna pediátrica viva atenuada (cepa Schwarz) contra el sarampión (MV). Como prueba de concepto, insertamos en el vector del sarampión un antígeno combinado del dengue compuesto del dominio III (EDIII) de la proteína de envoltura del dengue y del ectodominio de la proteína de membrana (ectoM) de DV-1. La inmunidad inducida por el virus recombinante MV-EDIII-ectoM fue evaluada *in vivo* en ratones sensibles al sarampión e *in vitro* en células dendríticas humanas (DCs). Los ratones inmunizados desarrollaron anticuerpos neutralizantes específicos de dengue 1. La presencia de ectoM fue necesaria para lograr inducir una respuesta inmune contre EDIII. La capacidad adyuvante del ectoM se correlacionó con su potencial de inducir la maduración de las DCs y de promover la secreción de citokinas pro inflamatorias y antivirales, y de chemokinas implicadas en el establecimiento de la inmunidad adaptativa específica. La capacidad protectora de esta vacuna merece ser evaluada en Primates-No Humanos. Una vacuna pediátrica combinada sarampión-dengue podría ser de particular interés para inmunizar niños simultáneamente contra el sarampión y el dengue en las zonas del mundo donde ambas enfermedades circulan.
