# Supplementary material for: Pediatric Measles Vaccine Expressing a Dengue Antigen Induces Durable Serotype-specific Neutralizing Antibodies to Dengue Virus
Source: PLoS Negl Trop Dis. 2007 Dec 12;1(3):e96. doi: 10.1371/journal.pntd.0000096 (PMC2154386; doi:10.1371/journal.pntd.0000096)
Supplement: Alternative Language Abstract S2 — Translation of abstract into French (0.02 MB DOC) [file pntd.0000096.s002.doc]

**RESUME (french)**

(translation of the abstract in French by author Frédéric Tangy)

La fièvre dengue est une maladie émergente qui menace le tiers de la population mondiale. Malgré des décennies d’efforts, aucun vaccin n'est encore disponible contre cette maladie virale transmise dans les régions tropicales par les moustiques. Dans le but de développer un vaccin accessible aux enfants et adolescents vivant dans les régions tropicales à risque, nous proposons une nouvelle stratégie basée sur l'expression d'un antigène minimal du virus de la dengue par un vecteur dérivé du vaccin vivant atténué pédiatrique contre la rougeole. Pour faire la preuve de concept de cette stratégie, nous avons inséré dans le vecteur rougeole (MV) une séquence codant pour un antigène combiné minimal du virus de la dengue composé du domaine III de la glycoprotéine d'enveloppe (EDIII) fusionné à l'ectodomaine de la protéine de membrane (ectoM) du virus de la dengue sérotype-1. L'immunogénicité du vaccin recombinant MV-EDIII-ectoM a été évaluée chez des souris transgéniques sensibles à l’infection par le virus de la rougeole, et in vitro sur des cellules dendritiques primaires humaines (DC). L'immunisation des souris a induit la production à long terme d’anticorps neutralisants DV1 spécifiques du sérotype-1. La présence de l'ectoM est déterminante pour l'immunogénicité du domaine EDIII. La capacité adjuvante de l'ectoM est corrélée avec sa capacité de maturer les DCs et de favoriser la sécrétion de cytokines proinflammatoires et antivirales ainsi que des chimiokines impliquées dans l'établissement de l'immunité adaptative spécifique. La capacité protectrice de ce candidat vaccin sera évaluée dans un modèle de primate. Une telle stratégie de vaccination combinée rougeole-dengue permettrait d’offrir des vaccins pédiatriques accessibles particulièrement attrayants pour immuniser les enfants simultanément contre la rougeole et la dengue dans des régions du monde où les deux maladies sévissent.
